# Female control of mate plugging in a female-cannibalistic spider (*Micaria sociabilis*)

**DOI:** 10.1186/s12862-014-0278-9

**Published:** 2015-02-13

**Authors:** Lenka Sentenská, Stano Pekár, Elisabeth Lipke, Peter Michalik, Gabriele Uhl

**Affiliations:** Department of Botany and Zoology, Masaryk University, Brno, Czech Republic; Department of General and Systematic Zoology, Zoological Institute and Museum, Ernst Moritz Arndt University of Greifswald, Greifswald, Germany

**Keywords:** Secretory plugs, Sperm competition, Cryptic female choice, Multiple mating, X-ray microscopy

## Abstract

**Background:**

Sperm competition imposes a strong selective pressure on males, leading to the evolution of various physiological, morphological and behavioral traits. Sperm competition can be prevented by blocking or impeding the access to female genitalia by means of a mating plug. We investigated the factors responsible for plug production and function in the promiscuous female-cannibalistic spider *Micaria sociabilis* (Gnaphosidae).

**Results:**

We performed mating trials using females with and without a plug that consists of an amorphous mass. The mating trials demonstrated that the probability of male plugging increased non-linearly with the duration of copulation. Copulation duration and plug production seem to be controlled by the female. We found that females terminated matings later if males were fast at genital coupling. Whereas incomplete plugs had disappeared on the day following copulation, complete plugs persisted (40%). In matings with females with complete plugs, only a small proportion of males (7%) were able to remove the plug, indicating the high effectiveness of plugging. Moreover, males ceased attempts to copulate with plugged females with higher probability. 3D X-ray microscopy of the female and male genitalia showed that the plug material can extend far into the female genital tract and that the plug material is produced by a massive gland inside the palpal organ of the modified male pedipalps.

**Conclusions:**

Our study demonstrates that the mating plug in *M. sociabilis* constitutes an effective male strategy to avoid sperm competition that seems to be under female control.

**Electronic supplementary material:**

The online version of this article (doi:10.1186/s12862-014-0278-9) contains supplementary material, which is available to authorized users.

## Background

In polyandrous mating systems, competition between males for fertilization success continues even after copulation. If two or more males copulate with a single female and their ejaculates temporally overlap, competition between spermatozoa for access to female eggs is expected [1,2].

Sperm competition represents a strong selective force shaping morphological, physiological and behavioural traits. As a result, males evolved a variety of strategies to cope with sperm competition, e.g. they may directly outcompete rivals, avoid sperm competition by means of displacing previously stored ejaculates from the female genital tract, or prevent the female from receiving sperm from subsequent males [2,3]. Males engage in post-copulatory mate guarding [2] or block female copulatory openings to physically impede subsequent copulations [4] to achieve the latter. Mating or copulatory plugs were demonstrated in a few species from a wide range of taxa including acanthocephalans [5], nematodes [6], arthropods (arachnids [7,8]; crustaceans [9,10]; insects [11,12]), reptiles [13,14] and mammals [15,16]; however, their effectiveness has rarely been studied. In spiders, mating plugs are widely distributed (reviewed in [17]), a situation which is facilitated by several morphological and behavioural aspects: females can store sperm from several males in their sperm storage organs (i.e. spermathecae) for a prolonged time; in entelegyne spiders, female copulatory openings are independent of the gonopore used for oviposition [18], thus plugging the openings does not interfere with oviposition. Female copulatory opening(s) can host parts of the male body or an amorphous material. In a considerable number of species, a part or the whole of the male copulatory organ is broken off during copulation and left inside the female genital tract [17]. Such genital plugs, which leave the palpal organ functionally sterile (but see [19]), were shown to prevent or reduce future inseminations in most species that have been investigated so far (e.g. [20-25];). In some species, even the whole male body may serve as a short term mating plug [26].

A less dramatic type of potential plugging is the production of an amorphous substance that is applied in the female copulatory openings. The properties and amount of the material in the female genital system differ among [17] as well as within species [27,28]. The material is suspected to be produced by glandular tissue situated in the male copulatory organ [29], male mouth area [30], or male genital tract [31]. While plugs formed by parts of a male copulatory organ can plug only one copulatory opening, amorphous plugs may seal both copulatory openings when female copulatory ducts are situated close to each other or open in a single cavity, as often seen in entelegyne spiders (for examples see [17]). Consequently, males may monopolize a female with a single insertion [17].

However, the production of long-lasting and resistant secretory plugs can strongly oppose female interests. Therefore, pronounced female mate choice is expected prior to plug production [17]. Theoretically, females can affect plug production and effectiveness by controlling the number of insertions or copulation duration [28,32], or by selectively adding a substance to the plug material transferred by a male that enhances hardening [33]. Since small or incomplete plugs can be overcome, males have to transfer a sufficient amount of plug material to plug the female effectively [34]. Plug size and effectiveness depend on various traits such as male size [34], copulation duration [32], and the age of the plug [28].

The goal of this study was to examine the frequency and effectiveness of genital plugging in the promiscuous female-cannibalistic spider *Micaria sociabilis* Kulczyński, in which males deposit an amorphous material on or in the female genitalia. There is no courtship, females struggle during male attempts to achieve a copulation posture, and sometimes they become victims of cannibalistic males [35,36]. We investigated the behavioural aspects of plugging and plug removal by means of mating experiments and conducted morphological studies of male and female genitalia to elucidate plug position and the origin of the plug material. In the first experiment we paired males with non-plugged females to investigate which behavioural parameters affect plugging. We hypothesize that the production of the material is affected by physical attributes of the male (i.e. size), with larger males being better at overcoming female resistance [37] and subsequently applying a plug. If plugging opposes female interests, we expect female mate choice during mating and female behaviour to affect the success of plug production. In the second experiment we performed mating trials with already plugged females to investigate the male ability to remove the plugs. Further, we compared matings with females with and without plugs to assess the effectiveness of mating plugs as mechanical barriers, an aspect that has largely been ignored in previous investigations of mating plugs. The morphology of the male genital bulb and the female genital system were investigated to reveal where the plug material is produced and how far it extends into the female copulatory ducts.

## Results

### Mating behaviour

After first contact, the males chased the vigorously moving females and tried to mount them. Spiders mated in the type III posture [18], meaning that the male approached the female from the front, climbed on the female prosoma, and reached around her opisthosoma to insert one of his pedipalps. His right palp was inserted into her left copulatory duct or vice versa. When the male achieved the correct mating position the female always continued to move, on average for (mean ± SE) 8.7 ± 1.9 minutes (N = 16). Attempts to couple the pedipalp to the female genital plate (i.e. genital coupling) were always accompanied by palpal chewing (the male moved a pedipalp between his chelicerae), brief pedipal coupling (i.e. flubs), and lateral palpal movements (for a description of these behaviours, see Additional file 1), all occurring repeatedly before the coupling of the pedipalp to the female genitalia. Once the pedipalp was coupled to the female genitalia, rhythmic full expansions of the membranous part (haematodocha) of the sperm transfer organ followed. During copulation, the pedipalp was coupled for an average of 77.7 ± 13.0 min (N = 16). Before mating ended, a sequence of brief haematodochal expansions was performed in 50% of cases (N = 16).

In 74.3% of cases (N = 35), copulation ended after a single insertion. Accordingly, both pedipalps were used in succession in only 25.7% of cases. The copulation ended when the female began to move vigorously or when the male left the female without any previous struggling. In the latter case we assume male driven termination. The sequential behaviours observed during mating trials are listed and defined in the Additional file 1.

### Mating trials with females without plugs

Descriptive data on the mating procedure with females without a previous plug are given in Table 1. Immediately after mating, a complete plug (i.e. covering the whole atrium) was found in 40% of cases (N = 35). In 20% of these cases an incomplete plug was found, i.e. plug material was found in the atrium in various amounts but it did not cover both copulatory openings. In the remainder of the cases (40%) no plug material was found in the atrium of the female. The plug material appeared no earlier than after 37 minutes of copulation. The copulation lasted on average (mean ± SE) 29.4 ± 19.7 min when no plug was produced, 97.9 ± 25.7 min when an incomplete plug was produced, and 137.1 ± 21.6 min when a complete plug was produced. Right after copulation the plug resembled a whitish, gelatinous substance. After one day, the substance had hardened and had the appearance of a black solid mass. One day after copulation, only complete plugs persisted in the female genital atrium, whereas incomplete ones had disappeared.

**Table 1:** **Descriptive data on separate mating trials with females without plugs and plugged females and statistical comparison of the mating behaviours between the two groups**

	**Mating trials with females**		**Statistical comparison**		
**Response variable**	**Without plug (N)**	**With plug (N)**	**Test statistics**	**P-value**	**Method/Model**
Succesful mounting [%]	53 (66)	49 (85)	*χ* ^2^_1_ = 0.07	0.78	Proportion test
Time in mating position (mean ± SE) [min]	86.2 ± 14.9 (35)	17.2 ± 3.4 (42)	F_1, 76_ = 39.2	**< 0.001**	GEE-gamma
Number of palps used for insertion (mean ± SE)	1.4 ± 0.1 (35)	1.07 ± 0.08 (42)	*χ* ^2^_1_ = 7.8	**0.01**	GEE-poisson
Number of lateral palpal movements per min (mean ± SE)	5.0 ± 1.2 (16)	22.4 ± 2.7 (24)	F_1, 39_ = 7.5	**0.006**	GEE-gamma
Number of palpal chewing movements per min (mean ± SE)	0.6 ± 0.1 (16)	2.0 ± 0.2 (24)	F_1, 39_ = 5.1	**0.024**	GEE-gamma
Presence of long haematodochal expansions [%]	100 (16)	8.3 (24)	*χ* ^2^_1_ = 3774	**< 0.001**	GEE-binomial
Copulation terminated by male [%]	2.9 (35)	52 (42)	*χ* ^2^_1_ = 7.9	**0.004**	GEE-binomial

Analyses of the video recordings (N = 16) revealed that plugs were probably produced by a sequence of brief haematodochal expansions at the end of the mating process. These expansions followed the period of distinct full haematodochal expansions and occurred shortly before genital uncoupling. The duration of one brief expansion was less than a second, while long haematodochal expansions lasted on average 2.6 ± 0.4 s (N = 16). The number of brief expansions in a sequence was on average 6.1 ± 1 (N = 16). Brief haematodochal expansions were observed in half of the video recorded trials (N = 16) and when these expansions occurred plug material was found in the female atrium (N = 8). In the rest of the trials (N = 8) no brief expansions were observed and no plug material was detected in the female atrium. The amount of plug material in the atrium was significantly affected by the presence of brief expansions (ANOVA, F_1, 14_ = 36.2, *P* < 0.001), but did not significantly depend on their number (Spearman’s r = -0.44, *P* = 0.27).

**Figure 1:**
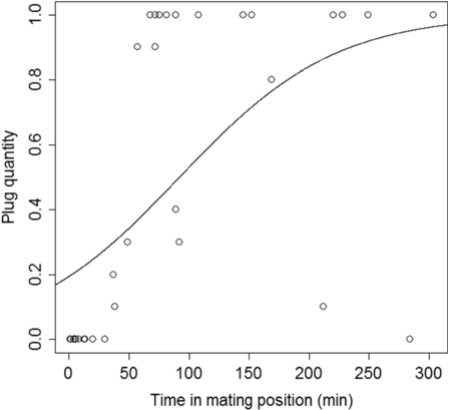
**Plug quantity (proportion of plug material in the female atrium) in relation to time in mating position.** Estimated logit model is shown.

**Figure 2:**
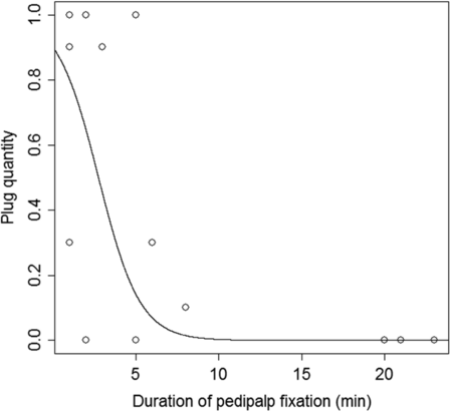
**Plug quantity (proportion of plug material in the female atrium) in relation to duration of palp fixation.** Estimated logit model is shown.

The amount of plug material found in the female atrium was related to increasing time in the mating posture (GLS, F_1, 33_ = 26.4, *P* < 0.001, Figure 1), which was negatively affected by female restlessness (GLM-g, F_1,14_ = 14.4, *P* = 0.001). Neither male size (GLS, F_1, 27_ < 0.1, P = 0.89) nor female size (GLS, F_1,27_ < 0.1, P = 0.99) nor the size ratio in the pair (GLS, F_1, 23_ = 0.04, P = 0.83) taken as PCA1 scores affected the occurrence or size of the plug. The amount of plug material was negatively affected by the duration of the genital coupling phase (N = 12, *r*_s_ = -0.77, P = 0.003, Figure 2). When males were able to couple their pedipalp quickly, a complete plug was found after mating. When they fixed the pedipalp later, plugs were incomplete or missing. After males had mounted the females, the females moved for 8.7 ± 1.0 minutes on average.

### Mating trials with plugged females

Descriptive data on the mating procedure with plugged females are given in Table 1.

Removal of a complete plug only occurred in 7% of cases (N = 42), and was not accompanied by conspicuous male behaviour. In two of these cases, full haematodochal expansions occurred after removal of the plug, followed by plug production in one case (N = 24). In the remaining cases, partial haematodochal expansions (i.e. flubs) were observed, which did not result in plug production.

As described above (in the previous section), females who had an incomplete plug in the atrium after copulation (N = 10) bore no plug after one day. In subsequent mating trials, after three days, the second males who achieved copulation (N = 9) used the same palp and thus the same copulatory duct as the previous male in 56% of cases and long haematodochal expansions were always performed.

### Comparison of mating trials with females with and without plugs

The behavioural components of the mating procedure observed in the experiments described above were compared between the two groups (Table 1). The probability of males mounting the female did not differ between trials with females with and without plugs. However, the length of time the couple remained in the mating posture was significantly shorter in plugged females than in females without a plug. When paired with plugged females, males performed significantly fewer insertions and performed significantly more lateral palpal movements and palpal chewing movements per minute than with females without plugs. Long haematodochal expansions occurred in all trials with females without plugs, but only in two cases in trials with plugged females and only after removal of the plug. In the rest of the cases (N = 22) only partial haematodochal expansions were observed (i.e. flubs).

**Figure 3:**
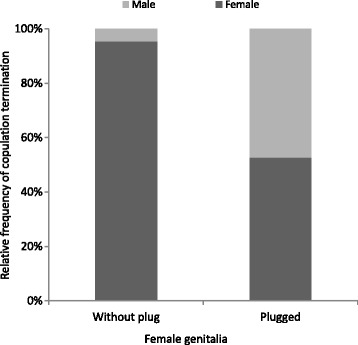
**Comparison of relative frequency of males and females terminating the copulation in experiments with females with and without plugs.**

Terminations of copulations with females without plugs were preceded by the female struggling vigorously, which resulted in the male being shaken off; only in one case did the male end the copulation (N = 35). Interactions with plugged females were ended by the female in 52% of cases (N = 42); in the remaining 48% of cases, males ceased attempting to fix their palp after 9.7 ± 1.5 minutes (N = 20) and left the females. Therefore, males were significantly more likely to end interactions when trying to mate with plugged females (Table 1, Figure 3).

### Male genitalia

**Figure 4:**
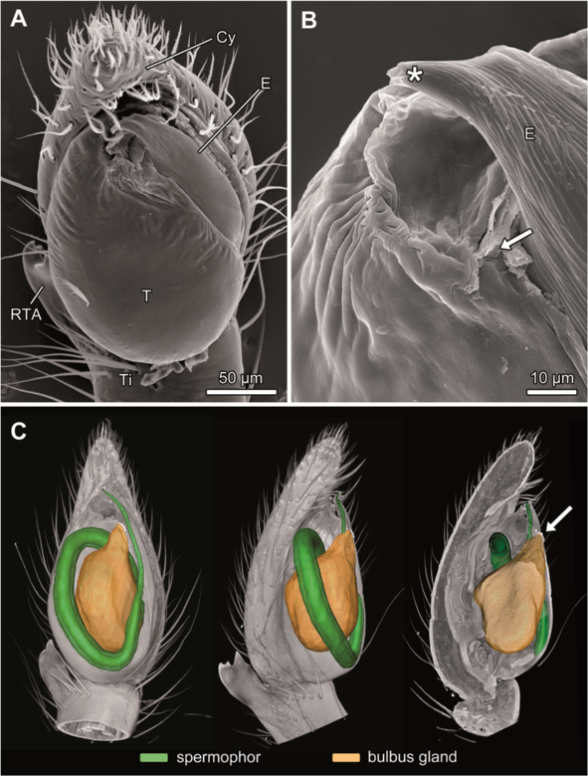
**Morphology of the male genital bulb of** ***M. sociabilis*** **. A** – **B**: SEM; **C**: Volume and surface reconstruction. **A**. Ventral view of genital bulb. **B**. Detail of the bulbus gland opening with remnants of plug material (arrow). Asterisk marks tip of the embolus bearing spermophor opening. **C**. Ventral and prolateral view of genital bulb and its longitudinal section. The gland reservoir encircled by loops of spermophor opens up below embolus (arrow). Cy – cymbium, E – embolus, RTA – retrolateral tibial apophysis, T – tegulum, Ti – tibia.

Males of *M. sociabilis* possess a relatively simple copulatory organ consisting of an apically tapered, spoon-like cymbium and an egg-shaped genital bulb (palpal organ) (Figure 4A). The actual sperm transfer structure, the embolus, projects from the genital bulb. The tip of the embolus is bent and bears an opening (Figure 4B) through which the ejaculate is released during copulation. The embolus opening connects to a closed tube, the spermophor, which is coiled inside the bulbus and in which the seminal fluid is stored (Figure 4C).

**Figure 5:**
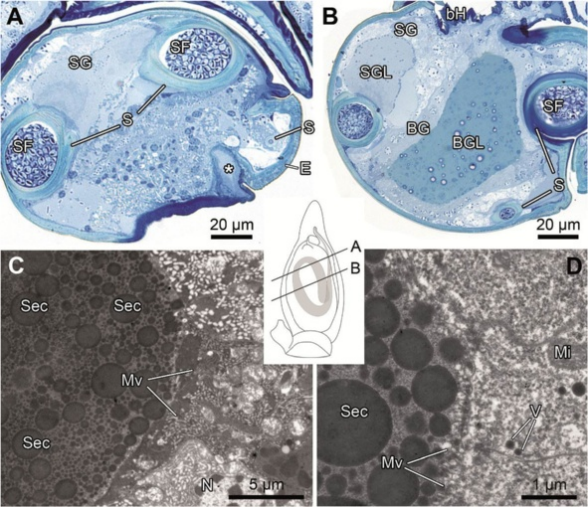
**Morphology of the male genital bulb of** ***M. sociabilis*** **. A** – **B**: LM; **C** – **D**: TEM. **A** – **B**: Semi-thin cross sections of the genital bulb showing duct **(A)** and reservoir **(B)** of the bulbus gland. Asterisk marks the homogenous plug material in duct lumen. **C** – **D**: Ultrathin section showing secretion in the lumen of the bulbus gland. BG – bulbus gland, BGL – bulbus gland lumen, bH – basal haematodocha, E – embolus, Mi – mitochondria, Mv – microvilli, N – nucleus, S – spermophor, Sec – secretion, SF – seminal fluid, SG – spermophor gland, SGL – spermophor gland lumen, V – vesicles.

Two glands were found in the genital bulb: a spermophor gland (Figures 5A, B) and a bulbus gland (Figures 4C, 5B, *sensu* Suhm et al. [29]). The spermophor gland is attached to the spermophor and runs along its wall. The massive bulbus gland takes a central position in the genital bulb, surrounded by the loops of the spermophor (Figures 4C, 5B). A single-layered epithelium surrounds the lumen of the bulbus gland (Figure 5B). Both glands exhibit numerous microvilli (Figures 5C, D). The suspected plug material is present inside the bulbus gland lumen.

The spermophor gland releases a secretion into the spermophor lumen through its porous wall. Even though the bulbus gland is surrounded by the spermophor, it is not connected to it (Figures 5A, B). The glandular lumen protrudes into a short duct which opens at the base of the embolus (Figures 4B, C). The opening is slit-like and covered by membranous folds of the apical part of the tegulum. The SEM investigation of the pedipalp revealed remnants of plug material covering the slit (Figure 4B).

### Female genitalia

**Figure 6:**
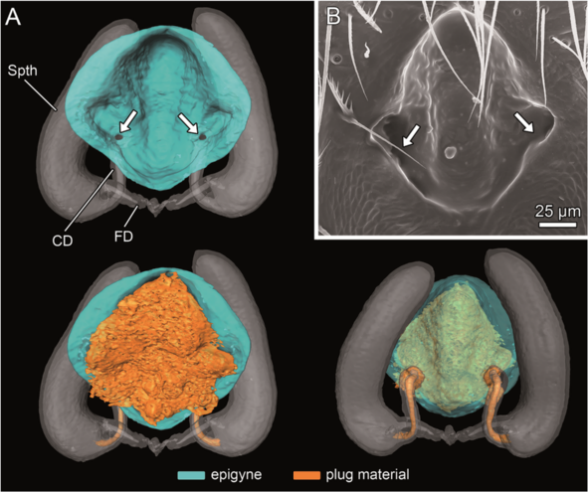
**Morphology of female genitalia of** ***M. sociabilis*** **. A**. Ventral and dorsal view of surface reconstruction of female epigynal plate with and without plug. **B**. SEM of female epigyne without plug. Arrows show copulatory openings. CD – copulatory duct, FD – fertilization duct, Spth - spermatheca.

**Figure 7:**
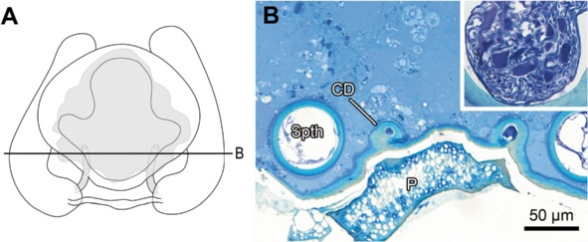
**Semi-thin cross sections of plugged female genitalia of** ***M. sociabilis*** **.** **A**. Schematic drawing of the female genitalia with a line showing the position of the cross section. **B**. Cross sections. Atrium and copulatory ducts are filled with plug material. Inset shows detail of spermatheca filled with seminal fluid. CD – copulatory duct, P – plug, Spth – spermatheca.

In females of *M. sociabilis*, the epigynal plate forms a trapezoidal cavity, an atrium, with two laterally-situated copulatory openings (Figures 6A, B). The openings lead to copulatory ducts, each connecting to a tubular spermatheca (Figure 6A). Near the proximal region of the copulatory duct, the fertilization duct leaves the spermatheca in the direction of the uterus. In all inspected plugged females, the plug material covered the whole atrium (N = 5 and N = 51 from the two experiments, respectively, Figure 6A) and extended also to both copulatory ducts up to the spermathecae, as revealed by X-ray microscopy (Figure 6A) and histology (Figure 7).

## Discussion

In species with polyandrous mating systems, males have evolved various strategies to cope with or avoid sperm competition. These strategies are determined by female morphology and the life history of the particular species [38]. In spiders, males generally experience a high risk of sperm competition because females can store fertile sperm after copulation for a prolonged time [39]. Although female genital morphology in entelegyne spiders is considered to favour sperm from the first male [39], but see [40], genital morphology in *M. sociabilis* rather favours sperm from the subsequent male, since both the copulatory and fertilization ducts connect to the spermatheca next to each other. Therefore, strategies opposing access to the female by a subsequent male, such as mate guarding or genital plugging can be expected. *Micaria sociabilis* is a cursorial spider actively searching for prey, and mate guarding has never been observed. Hence, plug production seems to be the optimal strategy to secure paternity. Indeed, we demonstrated that the material that is deposited in the female genital atrium after mating is a means of preventing re-mating in *M. sociabilis*.

A remarkable number of organisms engage in the production of mating plugs of various materials and effectiveness. In general, effective mating plugs counter polyandry and thereby may oppose female interests if females benefit from receiving more sperm or sperm from several males [41,42]. Consequently, females are expected to evolve adaptations either to counteract the plugging [21] or to take control over plug effectiveness. In *M. sociabilis*, the plug effectiveness increased with the increasing duration of mating. As mating is normally terminated by the female, plug effectiveness can be simply controlled by her. Since the transfer of plug material seems to occur during the period of brief haematodochal expansions, vigorous female movements leading to the end of copulation likely hinder the successful production of a complete plug. Incomplete plugs that did not cover the whole atrium did not persist after 24 hours. Furthermore, plug production was less likely to occur when females were restless during mating. As a consequence, only 40% of males produced a mating plug that survived 24 hours and had a high chance (93%) of withstanding the removal attempts of rival males.

Generally, cryptic female choice is expected to occur in species in which (1) females mate with more than one male, (2) the behavioural or physiological responses of females to males during or after copulation bias paternity success and (3) female responses are correlated with male morphological or behavioural traits [3]. Females of *M. sociabilis* mate multiple times and they exhibit different levels of restlessness during mating towards different males, which was found to affect plug production. The female response was not correlated to male size, as found in *Agelena limbata* Thorell [34]. Nevertheless, cryptic female choice is often affected by male behaviour such as courtship before copulation [43] and especially during copulation (i.e. copulatory courtship) [44]. Males of *M. sociabilis* do not engage in precopulatory courtship and we did not observe any behaviour resembling copulatory courtship. However, it seems that female choice is affected by male behaviour during genital coupling. We observed that, at the beginning of all mating trials, the female ran around the dish with the male that was trying to fix his pedipalp. Female restlessness seemingly hindered pedipalp fixation. Females ceased moving during mating in cases where males succeeded in fixing their pedipalp quickly. The probability of depositing plug material was significantly increased in these cases. It is possible that females examine male skills during the initial phase of mating and allow plug production accordingly.

The plug found in female genitalia in *M. sociabilis* consists of an amorphous material originating in the genital bulb of the male pedipalp. We found two glands in the genital bulb, as reported for the spiders *Amaurobius fenestralis* Stroem, *A. ferox* Walckenaer [29] and *Oedothorax retusus* Westring [8]: the spermophor gland and the bulbus gland. The bulbus gland is not connected to the spermophor but opens independently via a slit-like opening below the tip of the embolus, as was found in two *Amaurobius* species [29] (but see [8] for *O. retusus*). The separate openings facilitate the release of plug material independent of sperm transfer. The transfer of the material is likely achieved by the same mechanism underlying the expulsion of the sperm; that is, increasing haemolymph pressure [29], which causes expansions of the haematodochal membrane. During mating trials, three types of haematodochal expansion during pedipalp movements were distinguished – flubs (i.e. partial expansions connected with attempts to fix the pedipalp), full long expansions (very likely connected to sperm transfer), and full brief expansions. The latter occurred in rapid succession, always after the long expansions had stopped and shortly before the uncoupling of the pedipalp from female genitalia. When these brief pulses occurred, plug material was always found in the female atrium, indicating that they are responsible for the transfer of plug material. While in theridiid spiders the male has to reattach his pedipalp or even remount the female before transferring plug material [31], plug production immediately follows sperm transfer in *M. sociabilis*. The two processes are distinguishable only by differences in the duration of haematodochal expansions and it is possible that sperm transfer continues even during plug production. In *O. retusus*, plug production occurred without any conspicuous change in palpal organ movements [32], which is possibly because plug production occurs after sperm transfer via the spermophor, as the plug is transferred through the same opening as sperm [8].

It is assumed that secretory plugs impose a relatively low cost on a male compared to plugs composed of male genital parts, the use of which strongly reduces the occurrence of future matings [17,20,44] but see [25,45]. Due to costs connected with genital mutilation, plugs consisting of male genital parts are expected to exhibit higher effectiveness than amorphous plugs; however, both types of plug can be overcome [17]. The effectiveness of the mating plug is determined by its properties and female genital morphology. While genital plugs do not allow the male to plug both copulatory openings with a single insertion, both copulatory ducts can be plugged by a single large mass of secretion provided that both copulatory openings lie in close vicinity within an atrium. This is the case with *M. sociabilis* and our morphological study of its female genitalia revealed that the plug material not only fills the female atrium but also extends far into the copulatory ducts. Behavioural observations demonstrated that plugs in *M. sociabilis* indeed represent effective devices preventing females from engaging in subsequent copulation. Males paired with plugged females were scarcely able to remove the plug (7%) or copulate (4.7%). Males confronted with a complete plug performed significantly more lateral palpal movements and palpal chewing than when mating with females without plugs, such movements likely connected with attempts to remove the plug and fix the palp. However, it remains unclear whether males perform these behaviours to remove the plug or whether they simply try to fix their palp and the plug removal is a by-product of this effort. Moreover, while matings with females without plugs were terminated mainly by the female, matings with plugged females were terminated in half of the cases by males. Therefore, the time spent in the mating position was significantly shorter with plugged females. However, although males are likely selected to produce effective plugs, selection should simultaneously favour the male ability to remove the plugs of rivals in order to gain access to female ova, since a male will encounter plugged females with a high probability. These two counteracting forces result in situation in which 100% effective plugs cannot be stable over evolutionary time [46]. Indeed, a small proportion of males were able to displace the complete plug and some achieved full haematodochal expansions indicating sperm transfer. The probability of plug removal is particularly high in cases where plugs are incomplete, as has been shown for other spider species (Salticidae: [27], Agelenidae: [34]). In *M. sociabilis*, incomplete plugs did not even persist for 24 hours without any male interference. If the plug material is removed from the atrium, it is questionable whether the plug material inside the copulatory ducts can be removed as well or remains an obstacle to rival males. However, males often inserted their embolus into the copulatory duct of a female that previously had an incomplete plug. Since these males performed full long haematodochal expansions that indicate sperm transfer, we assume the copulatory ducts were accessible in these cases. Therefore, we assume that ducts are effectively plugged only when a complete plug is present. We did not investigate the removal of fresh plugs, but assume that it is easier to remove them as long as they are still viscous. In *O. retusus*, plugs serve as effective mechanical obstacles but their efficacy depends on their size and age. In this case, not fully hardened plugs can be removed or overcome easily by subsequent males [28].

## Conclusions

We found that males of *M. sociabilis* produce mating plugs, the material for which originates in a gland in the male pedipalp. Plugs are effective only when males produce a sufficient amount of plug material to fill the whole atrium. Only males capable of early genital coupling copulated long enough to produce a complete plug. Since the duration of mating was strongly affected by female behaviour, we presume that plug production is at least partly under female control. Even though males approached and mounted plugged females, plug removal was very rare. In conclusion, complete mating plugs in *M. sociabilis* represent a reliable device preventing sperm competition.

## Methods

Adult and subadult individuals of *M. sociabilis* were collected with a pooter from the tree bark of dozens of trees in Lednice, Czech Republic from March to August in 2012. Spiders were housed individually in glass tubes (diameter 15 mm, length 60 mm, average spider body size approx. 3.2 mm) with a layer of gypsum on the bottom, which was moistened with few drops of water at 2-day intervals to maintain the required humidity. Spiders were kept at room temperature (approx. 22°C), at 40% RH, and under a natural LD regime and were fed with fruit flies (*Drosophila melanogaster* Meigen) and springtails (*Sinella curviseta* Brook) to satiation at 2-day intervals and a day before morphological studies and behavioural experiments were performed.

### Analysis of mating behaviour

Females collected from the field were anesthetised with CO_2_ and their genital area was checked for the presence of a plug under a stereomicroscope (Olympus SZX9) at × 50 magnification. If no plug was apparent, they were used in the experiment investigating plug production (see below). Females with plugs were used to examine plug removal probability (see below). Some males and females were used up to three times in the mating trials (see below). The interval between matings was a minimum of 3 days.

All mating trials (N = 151, females without plug N = 66, plugged females N = 85) were performed in the laboratory between March and August 2012. All trials were carried out in Petri dishes (diameter 35 mm, height 10 mm). Observations of mating were performed under a stereomicroscope (Olympus SZ61) at × 4.5 magnification and with a mirror placed under the dish. After each trial, the dish was cleaned with 70% ethanol and allowed to air dry before re-use. At the beginning of a trial, first the female and then the male were released into the dish. If the male did not mount the female within 20 minutes of first contact, the trial was terminated. If the male mounted the female, the time in the mating position (used as a proxy of copulation duration) and the number of pedipalp insertions was recorded. Further, the sex that terminated mating was registered on the basis of whether the female’s vigorous movement preceded the termination (categorised as terminated by the female) or not (categorised as terminated by the male).

Forty mating trials (females without plug N = 16, plugged females N = 24) were recorded using a video recorder (SONY GV-HD700 HDV) connected to a colour camera (CV-735) attached to stereomicroscope (Olympus SZ61) at × 4.5 magnification. In these trials, the following behaviours were analysed: the number of palpal chewing movements the male performs per minute while trying to couple his genitalia; the number of lateral palpal movements performed per minute during genital coupling; the genital coupling duration (i.e. the time interval from mounting the female until palp fixation); the presence of long haematodochal expansions after palp fixation; and the presence and number of brief haematodochal expansions occurring shortly before the uncoupling of the palp (see Additional file 1 for definitions). The occurrence of long haematodochal expansions during copulation was considered as a proxy for sperm transfer [18]. Further, female restlessness (i.e. the proportion of the time the female moved during mating) was recorded. The prosoma length and width of all individuals were measured using an ocular micrometer on a stereomicroscope (Olympus SZ61).

The description of the mating is based on trials with females without plugs (N = 35, section Mating behaviour), as plug presence may affect the normal mating procedure. In all these trials we noted the number of pedipalp insertions and the sex of the individual terminating copulation. Detailed description (the number of palpal chewing movements, the number of lateral palpal movements, the duration of genital coupling, the presence of long haematodochal expansions, and the presence and number of brief haematodochal expansions) is based on the video recorded trials (N = 16).

### Mating trials with females without plugs

Adult males (N = 40) collected in the field were paired with adult females without plugs, which were collected either as adults (N = 24) or subadults (N = 10) that were reared to adulthood under laboratory conditions as described above. Before the females were used in mating trials, their genital area was photographed at × 50 magnification using a Leica DFC290 Digital Camera attached to an Olympus SZX9 stereomicroscope. When the male managed to mount the female and contacted her genital plate, the female genitals were checked and photographed again immediately after the mating trial and again one day later. When a plug was present, the extent to which it covered the atrium was estimated in percent. Altogether 66 trials were conducted. Some individuals were used repeatedly in the mating trials (18 males and 8 females were used once, 18 males and 20 females were used twice, and 4 males and 6 females were used three times). In 35 mating trials males successfully mounted the female and 16 of these trials were video recorded and analysed for the behaviours described above. The trials and individuals used in them were randomly selected and the individuals were used only once. We could not determine whether the adult females were virgins or not; however, as we collected adult females at the beginning of the season, we assume that we sampled mostly virgin females. None of the measured components of mating (time in mating position, female restlessness, female size, duration of genital coupling phase, no. of palps used, no. of lateral palpal movements, and no. of palpal chewing movements) differed between females collected in the field as adults (unknown mating status) and virgin females which moulted in the laboratory (*P* > 0.21). Further, mated but unplugged females were observed to re-mate with subsequent males under laboratory conditions. Therefore, we pooled the data and analysed all females together. To investigate whether the brief haematodochal expansions occurring at the end of copulation are linked to plug production, the amount of plug material in relation to the presence/absence of brief expansions was analysed using ANOVA. The relative amount of plug material was transformed using angular transformation in order to homogenize variances. Spearman’s rank correlation was used to test for an association between the relative amount of plug material in the atrium and the number of brief pulses and genital coupling duration. The effect of time spent in the mating position on plug production was analysed using Generalised Least Squares (GLS) with the AR(1) autoregressive structure due to repeated measurements [47]. GLS is an extension of the General Linear Model that produces a marginal model. The non-independence covariance structure in residuals is then used to produce correct SE values of parameter estimates as well as correct inferences. In this and in the following analyses, the relative amount of plug material was again transformed using angular transformation to homogenize variance. The effect of female restlessness on time spent in the mating position was analysed using the Generalised Linear Model (GLM) with Gamma error structure and log link (GLM-g). The body size of males and females (the length and width of the prosoma were used as predictors) and the size ratios in the pair (male:female) were all correlated; thus the data were subjected to Principal Component Analysis (PCA) in order to extract scores along the first axis. The scores were then used as an auxiliary explanatory variable in analysis using Generalised Least Squares (GLS) with the AR(1) autoregressive structure.

### Mating trials with plugged females

Adult males (N = 57) collected in the field were paired with adult females with complete plugs (i.e. covering the whole atrium; N = 51). Females were anesthetised with CO_2_ before and after the mating trial in order for pictures of their external genitalia to be taken. Some individuals were used repeatedly in the mating trials, up to three times (31 males and 18 females were used once, 24 males and 32 females were used twice, and 2 males and 1 female was used three times). 24 mating trials with individuals that were used only once were videotaped and analysed as stated above.

Females from the experiment on plug production that exhibited incomplete plugs in the atrium following copulation (N = 10) were re-mated after three days to study the effectiveness of the incomplete plugs. If the second males managed to mount the female, we recorded the presence of full haematodochal expansions and which palp was used.

### Comparison of mating with females with and without plug

To clarify the effect of plug presence on mating behaviour, several parameters were compared between the trials with females without plugs from the two experiments (Table 1). The numbers of males mounting the female in trials with females with and without plugs were compared using the Proportion test. Individual behavioural components of mating were analysed using Generalized Estimation Equations (GEE) with Gamma error structure and log link (the number of lateral palpal movements during the coupling phase per minute, the number of palpal chewing movements per minute, time spent in the mating position), Poisson error structure and log link (number of palps used), and binomial error structure and logit link (sex of the individual terminating the copulation). GEE is an extension of GLM that can handle data with some sort of correlation that, in this study, arose due to repeated use of some individuals in the mating trials. Similarly to GLS, GEE produces marginal models with correct SE estimates and inferences. The association structure used was autoregressive AR(1) due to repeated measurements [49].

All statistical analyses were performed in R environment [48]. Descriptive statistics are given as arithmetic mean ± standard error.

### Morphology of male and female genitalia

For scanning electron microscopy, two female opisthosomata (one with, and one without a plug in the genital area) and three male pedipalps from specimens preserved in ethanol were critical point dried, mounted, coated with gold, and studied under a JEOL JSM-6380LV.

For histological and ultrastructural analysis, female opisthosomata with a plug in the genital area (N = 3) and male pedipalps (N = 4) were fixed in 2.5% glutaraldehyde in phosphate buffer (0.1 M, pH 7.2, 1.8% sucrose), then subject to postfixation in buffered 2% osmium tetroxide. The samples were then washed in phosphate buffer, dehydrated in graded ethanols, and embedded in SPI-PON 812 resin. Serial semi-thin sections (700 nm, cross sections) of male pedipalps and female opisthosomata were obtained using a Diatome Ultra 45° diamond knife on a Leica ultramicrotome UC6 and stained according to Richardson et al. [49]. Examination of the semi-thin sections was performed with an Olympus BX60 connected to a Zeiss MCr digital camera. Ultrathin sections (60 nm, cross sections) of male pedipalps and female genital regions were placed on copper slot grids (Science Services) and covered with a thin layer of desiccated pioloform solution (1% Pioloform in 100% Chloroform). Post-processing included staining with saturated uranyl acetate and lead citrate according to Reynolds [50], using a PELCO SynapTek gridStick. The sections were examined with a transmission electron microscope (TEM) JEOL JEM 1011 at 80 kV. Images were obtained with an Olympus Mega View III digital camera using iTEM software.

### 3D X-ray microscopy (XRM) and 3D reconstruction

After fixation (see protocol above), a female opisthosoma with plugged genital openings and three male pedipalps were prepared for an X-ray microscopy scan (Xradia XCT-200, Carl Zeiss Microscopy GmbH) in the following way: after dehydration, samples were stained overnight using 1% iodine solution (in pure ethanol). After washing in pure ethanol, samples were critical point dried (BalTec 30) and subsequently mounted on insect pins using super glue. Scans were then obtained using the 40x objective lens unit with the following scan parameters: 40 kV, 8 W, 200 μA, exposure time 30 sec/frame. Reconstructed image stacks were created using XMReconstructor software (Carl Zeiss Microscopy GmbH) and the subsequent segmentation (delineation) of the structures of interest in the male and female genitalia was performed with Amira 5.4.5 (Visualization Science Group, FEI). Measurements were obtained using the 3D length and volume measurement tool in Amira 5.4.5 (Visualization Science Group, FEI) and transformed to actual sizes based on the pixel to μm ratio.

## Additional file

Additional file 1:
**Description of sequential behaviours recorded during mating trials in** ***M. sociabilis.***

## References

[1] Parker GA (1970). Sperm competition and its evolutionary consequences in the insects. Biol Rev.

[2] Wigby S, Chapman T (2004). Sperm competition. Curr Biol.

[3] Eberhard WG (1996). Female control: sexual selection by cryptic female choice.

[4] Simmons LW (2001). Sperm competition and its evolutionary consequences in the insects.

[5] Dezfuli BS, Capuano S, Pironi F, Mischiati C (1999). The origin and function of cement gland secretion in *Pomphorhynchus laevis* (Acanthocephala). Parasitology.

[6] Barker DM (1994). Copulatory plugs and paternity assurance in the nematode *Caenorhabditis elegans*. Evol Heal Dis.

[7] Contreras-Gorduño J, Peretti AV, Córdoba-Aguilar A (2006). Evidence that mating plug is related to null female mating activity in the scorpion *Vaejovis punctatus*. Ethology.

[8] Uhl G, Kunz K, Vöcking O, Lipke E. A spider mating plug: origin and constraints of production. Biol J Linn Soc. 2014;113:345–54.

[9] Bauer RT, Min LJ (1993). Spermatophores and plug substance of the marine shrimp *Trachypenaeus similis* (Crustacea: Decapoda: Penaeidae): formation in the male reproductive tract and disposition in the inseminated female. Biol Bull.

[10] Oh SJ, Hankin DG (2004). The sperm plug is a reliable indicator of mating success in female Dungeness crabs, *Cancer magister*. J Crustac Biol.

[11] Baer B, Morgan ED, Schmid-Hempel P (2001). A nonspecific fatty acid within the bumblebee mating plug prevents females from remating. Proc Natl Acad Sci U S A.

[12] Polak M, Wolf LL, Starmer WT, Barker JSF (2001). Function of the mating plug in *Drosophila hibisci* bock. Behav Ecol Sociobiol.

[13] Shine R, Olsson MM, Mason RT (2000). Chastity belts in gartersnakes: the functional significance of mating plugs. Biol J Linn Soc.

[14] Moreira PL, Birkhead TR (2004). Copulatory plug displacement and prolonged copulation in the Iberian rock lizard (*Lacerta monticola*). Behav Ecol Sociobiol.

[15] Martan J, Shepherd BA (1976). The role of the copulatory plug in reproduction of the guinea pig. J Exp Zool.

[16] Dixson AL, Anderson MJ (2002). Sexual selection, seminal coagulation and copulatory plug formation in primates. Folia Primatol.

[17] Uhl G, Nessler SH, Schneider JM (2010). Securing paternity in spiders? a review on occurrence and effects of mating plugs and male genital mutilation. Genetica.

[18] Foelix R (2011). Biology of spiders.

[19] Snow LSE, Abdel-Mesih A, Andrade MCB (2006). Broken copulatory organs are low-cost adaptations to sperm competition in redback spiders. Ethology.

[20] Fromhage L, Schneider JM (2006). Emasculation to plug up females: the significance of pedipalp damage in *Nephila fenestrata*. Behav Ecol.

[21] Nessler SH, Uhl G, Schneider JM (2007). Genital damage in the orb-web spider *Argiope bruennichi* (Araneae: Araneidae) increases paternity success. Behav Ecol.

[22] Kuntner M, Kralj-Fišer S, Schneider JM, Li D (2009). Mate plugging via genital mutilation in nephilid spiders: an evolutionary hypothesis. J Zool.

[23] Knoflach B, Van Harten A (2001). Tidarren argo sp. nov. (Araneae: Theridiidae) and its exceptional copulatory behaviour: emasculation, male palpal organ as a mating plug and sexual cannibalism. J Zool.

[24] Fromhage L, Elgar M, Schneider J (2005). Faithful without care: the evolution of monogyny. Evolution (N Y).

[25] Zimmer SM, Welke KW, Schneider JM (2012). Determinants of natural mating success in the cannibalistic orb-web spider *Argiope bruennichi*. PLoS One.

[26] Foellmer MW, Fairbairn DJ (2003). Spontaneous male death during copulation in an orb-weaving spider. Proc R Soc London Ser B Biol Sci.

[27] Jackson RR (1980). The mating strategy of *Phidippus johnsoni* (Araneae, Salticidae): II. sperm competition and the function of copulation. J Arachnol.

[28] Kunz K, Witthuhn M, Uhl G (2014). Do the size and age of mating plugs alter their efficacy in protecting paternity?. Behav Ecol Sociobiol.

[29] Suhm M, Thaler K, Alberti G (1996). Glands in the male palpal organ and the origin of the mating plug in *Amaurobius* species (Araneae: Amaurobiidae). Zool Anz.

[30] Braun R (1956). Zur Biologie von *Teutana triangulosa* (Walck.) (Araneae; Theridiidae, Asageneae). Zeitschrift Für Wissenschaftliche Zool.

[31] Knoflach B (2004). Diversity in the copulatory behaviour of comb-footed spiders (Araneae, Theridiidae). Denisia.

[32] Uhl G, Busch M (2009). Securing paternity: mating plugs in the dwarf spider *Oedothorax retusus* (Araneae: Erigoninae). Biol J Linn Soc.

[33] Aisenberg A, Eberhard WG (2009). Female cooperation in plug formation in a spider: effects of male copulatory courtship. Behav Ecol.

[34] Masumoto T (1993). The effect of the copulatory plug in the funnel-web spider, *Agelena limbata* (Araneae: Agelenidae). J Arachnol.

[35] Sentenská L, Pekár S (2013). Mate with the young, kill the old: reversed sexual cannibalism and male mate choice in the spider *Micaria sociabilis* (Araneae: Gnaphosidae). Behav Ecol Sociobiol.

[36] Sentenská L, Pekár S (2014). Eat or not to eat: reversed sexual cannibalism as a male foraging strategy in the spider *Micaria sociabilis* (Araneae: Gnaphosidae). Ethology.

[37] Maklakov AA, Bilde T, Lubin Y (2004). Sexual selection for increased male body size and protandry in a spider. Anim Behav.

[38] Smith RL (1984). Sperm competition and the evolution of animal mating systems.

[39] Eberhard W (2004). Why study spider sex: special traits of spiders facilitate studies of sperm competition and cryptic female choice. J Arachnol.

[40] Austad SN, Smith RL (1984). Evolution of sperm priority patterns in spiders. Sperm compet evol anim mating syst.

[41] Watson PJ (1991). Multiple paternity as genetic bet-hedging in female sierra dome spiders, *Linyphia litigiosa* (Linyphiidae). Anim Behav.

[42] Jennions MD, Petrie M (2000). Why do females mate multiply? a review of the genetic benefits. Biol Rev Camb Philos Soc.

[43] Crews D (1987). Courtship in unisexual lizards: a model for brain evolution. Sci Am.

[44] Eberhard W (1994). Evidence for widespread courtship during copulation in 131 species of insects and spiders, and implications for cryptic female choice. Evolution (N Y).

[45] Miller JA (2007). Repeated evolution of male sacrifice behavior in spiders correlated with genital mutilation. Evolution.

[46] Fromhage L (2012). Mating unplugged: a model for the evolution of mating plug (dis-)placement. Evolution (N Y).

[47] Pekár S, Brabec M (2012). Modern analysis of biological data. 2. linear models with correlation in R.

[48] R Development Core Team. R: a Language and Environment for Statistical Computing. Vienna: R Foundation for Statistical Computing. 2010. http://www.Rproject.org.

[49] Richardson KC, Jarret L, Finke EH. Embedding in epoxy resins for ultrathin sectioning in electron microscopy. Stain Technol. 1960;35:313–23. 10.3109/10520296009114754 13741297

[50] Reynolds ES. The use of lead citrate at high pH as an electron-opaque stain in electron microscopy. J Cell Biol. 1963;17:208–12. 10.1083/jcb.17.1.208 PMC2106263 13986422

