# Supplementary material for: Female control of mate plugging in a female-cannibalistic spider (Micaria sociabilis)
Source: BMC Evol Biol. 2015 Feb 13;15(1):18. doi: 10.1186/s12862-014-0278-9 (PMC4327802; doi:10.1186/s12862-014-0278-9)
Supplement: Additional file 1: — Description of sequential behaviours recorded during mating trials in M. sociabilis. [file 12862_2014_278_MOESM1_ESM.pdf]

Behaviour	Description
**Mounting**	Male chases the female and tries to mount her to reach a mating posture. Male approaches the female from the front and tries to climb on her prosoma. Female vigorously moves during male attempts to mount.
**Mating position**	Male mounted the female and the pair is in a mating position, i.e. male is positioned dorsally by the side of the female so her epigynal plate is within a reach of male pedipalps.
**Genital coupling**	Once in mating position, male tries to couple one of his pedipalps with female genitalia. Attempts to couple the palp are accompanied by palpal chewing, flubs and lateral palpal movements.
**Palpal chewing**	Male moves his pedipalp between chelicerae.
**Flubs**	Male briefly couples his pedipalp with the female genitalia with short partial expansion of a haematodochal membrane. Pedipalp is detached after few seconds. During these expansions sperms are probably not transferred.

**Lateral palpal movements**	Male repeatedly moves his pedipalp below the epigyne without expansions of haematodocha.
**Pedipalp fixation**	After period of genital coupling, male fixes his pedipalp to female genitalia in right position for sperm transfer. Pedipalp remains fixed until the end of the copulation. Palpal chewing, flubs and lateral palpal movements are not occurring anymore.
**Copulation**	Male performs one or several palpal insertions with full haematodochal expansions. Insertion starts when palp is fixed and terminates when the palp is uncoupled from female genitalia.
**Long haematodochal expansion**	Full rhythmic expansions of haematodochal membrane connected with sperm release from the palp. One expansion lasts several seconds.
	Rapid succession of brief but full expansions of haematodochal membrane occurring after the sperm transfer (i.e. after long haematodochal expansions) and shortly before uncoupling the pedipalp from genitalia. One expansion lasts less than one second.
